# Association of fibrinogen level with early neurological deterioration among acute ischemic stroke patients with diabetes

**DOI:** 10.1186/s12883-017-0865-7

**Published:** 2017-05-19

**Authors:** Seong-Joon Lee, Ji Man Hong, Sung Eun Lee, Dae Ryong Kang, Bruce Ovbiagele, Andrew M. Demchuk, Jin Soo Lee

**Affiliations:** 1 Department of Neurology, Ajou University School of Medicine, Ajou University Medical Center, San 5, Woncheon-dong, Yeongtong-gu, Suwon, Kyungki-do 443–721 South Korea; 2 Center of Biomedical Data Science/ Institute of Genomic Cohort, Yonsei University Wonju College of Medicine, Wonju, South Korea; 3 Department of Neurology, Medical University of South Carolina, Charleston, SC USA; 4 Calgary Stroke Program, Departments of Clinical Neurosciences and Radiology, Hotchkiss Brain Institute, University of Calgary, Calgary, AB Canada

**Keywords:** Cerebral infarction, Disease progression, Diabetes mellitus, Fibrinogen, Intracranial thrombosis

## Abstract

**Background:**

Diabetes mellitus (DM) is a risk factor for early neurological deterioration (END) in acute ischemic stroke. The prothrombotic protein fibrinogen is frequently elevated in patients with diabetes, and may be associated with poorer prognoses. We evaluated whether fibrinogen is associated with END in patients with diabetes after acute ischemic stroke.

**Methods:**

We included 3814 patients from a single hospital database admitted within 72 h of onset of ischemic stroke. END was defined as an increase in the National Institutes of Health Stroke Scale (NIHSS) ≥2 within 7 days post-admission. In the total population (END, *n* = 661; non-END, *n* = 3153), univariate and multivariate analyses were performed to assess fibrinogen as an independent predictor for END. We then performed propensity score matching and univariate analyses for DM (END, *n* = 261; non-END, *n* = 522) and non-DM populations (END, *n* = 399; non-END, *n* = 798). Multiple logistic analyses were performed after matching for fibrinogen as a risk factor in each subgroup.

**Results:**

Fibrinogen levels were higher in the END group than in the non-END group (367 ± 156 mg/dL vs. 347 ± 122 mg/dL, *p* = 0.002), though they were not associated with END in logistic regression analyses. Fibrinogen levels were found to be an independent predictor for END, but only in the DM population (fibrinogen levels 300–599 mg/dL, odds ratio: 1.618, 95% confidence interval: 1.037–2.525, *p* = 0.034, fibrinogen levels ≥600 mg/dL, 2.575, 1.018–6.514, *p* = 0.046; non-DM population, *p* = 0.393). The diabetes-fibrinogen interaction for the entire cohort was *p* = 0.101.

**Conclusions:**

Elevated fibrinogen is dose-dependently associated with END in patients with diabetes following acute ischemic stroke.

**Electronic supplementary material:**

The online version of this article (doi:10.1186/s12883-017-0865-7) contains supplementary material, which is available to authorized users.

## Background

Diabetes mellitus (DM) and hyperglycemia negatively affect the outcome and pathophysiology of acute ischemic stroke. The associations between DM and early neurological deterioration (END) [1, 2], more comorbidities [3], severe handicap and disability [4, 5], and increased mortality [3], have been repeatedly reported in both case-control [6] and cohort studies [7]. When the effects of hyperglycemia are included, these patients also present with more severe lesion size [8], more symptomatic intracranial hemorrhages [9, 10], edema [11], and mortality [12]. Although END is more frequently encountered in diabetic patients, the specific mechanisms underlying how DM promotes END are among the least clear. In clinical practice, being able to anticipate END would allow for more timely intervention; thus, gaining further insight into the mechanisms of END specific to patients with DM would improve the ability to identify and intervene in END in a population seemingly more susceptible to it. One possible mechanism linking DM with adverse outcomes of stroke involves fibrinogen, a prothrombotic protein, and acute phase reactant that is consistently elevated in patients with diabetes [13, 14]. In fact, a large body of evidence identifies fibrinogen as a mediator in the development of coronary artery thrombi and future cardiac events [15, 16]. However, the association of hyperfibrinogenemia and END in acute ischemic stroke has not yet been demonstrated. In this study, we evaluated whether elevated fibrinogen levels in patients with diabetes and acute ischemic stroke are associated with END in a large, single-center population.

## Methods

### Study population

We performed a retrospective study that was approved by the Institutional Review Board, and performed in accordance with the ethical standards of the 1964 Declaration of Helsinki and its later amendments, incorporating consecutive patients enrolled in a hospital stroke database. From this database, we reviewed consecutive patients from Jan. 2000 to Dec. 2015. Among them, we included 3814 patients with acute ischemic stroke within 72 h of onset, and with available National Institutes of Health Stroke Scale (NIHSS) data.

### Data acquisition

For this study, the diagnosis of DM was defined as either a prior diagnosis of diabetes, or glycated hemoglobin (HbA1c) levels >6.1% at admission, according to a previous study which showed that prediabetic levels of hba1c >6.1% were associated with increased stroke recurrence [17]. Lab data concerning the hemostatic profiles such as fibrinogen, fibrin degradation products (FDP), D-dimer, and classic stroke risk factors were obtained on the day of admission, before initiation of intravenous tissue plasminogen activators for those who were applicable. Acute inflammatory markers, erythrocyte sedimentation rate (ESR), and standard C-reactive protein (CRP) levels were included in the analysis, to compare with fibrinogen. Components of metabolic syndrome were also analyzed. Lab data routinely taken to evaluate diabetic status, such as fasting glucose levels, urine glucose, urine protein, and HbA1c levels were also included in the analysis. Trial of Org 10,172 in Acute Stroke Treatment (TOAST) classification, and NIHSS scores on admission and at discharge were also collected from the database.

### Propensity score matching and statistical analysis

END was defined as a NIHSS score increase of 2 or more within 7 days after admission. A number of studies have, for the definition of END, used an increase of ≥2 points in the total NIHSS score within 7 days [18], as modified from a previous study. We have incorporated such sensitive criteria to define END for such subtle changes call for action in clinical practice. Because END in patients with acute ischemic stroke can be influenced by a greater initial NIHSS and TOAST classification, we used propensity score matching to adjust for well-known confounding factors. First, among the 3814 patients with acute stroke, we performed univariate and multivariate analyses to document the effects of fibrinogen and other predictive factors of END. Multivariate analysis was performed, adjusting for age, sex, presence of hypertension, diabetes mellitus, initial NIHSS score, and TOAST classification. The *p* value for the diabetes status x fibrinogen level interaction was also calculated in the logistic regression model. For subgroup analysis, the patients were divided into DM (*n* = 1360) and non-DM (*n* = 2454) populations. In each subgroup, a 1:2 propensity score matching of END patients and non-END patients was performed, adjusting for age, sex, initial NIHSS score, and TOAST classification (Additional file 1: Figure S1A-B). Differences between the two groups were analyzed before and after propensity score matching, using a χ^2^ test for categorical variables and Student’s *t*-test for continuous variables. Multiple logistic regression analysis was performed, adjusting for baseline clinical variables known to be associated with END such as age, sex, hypertension, initial NIHSS, and TOAST classification, as well as with potentially significant factors determined in the univariate analysis (*p* < 0.005), to verify the significance of fibrinogen levels on END. All statistical analyses were performed using IBM SPSS Statistics 20 software (Chicago, IL).

## Results

Among 3814 patients with acute stroke, 661 cases (17.3%) experienced early neurological deterioration. Patients in the END group were significantly older (65.3 ± 12.6 vs. 63.5 ± 13.5, *p* = 0.001), and more frequently had a history of DM (39.6% vs. 34.8%, *p* = 0.019), a history of hypertension (70.0% vs. 65.8%, *p* = 0.039), a higher initial NIHSS (8.2 ± 6.7 vs. 5.6 ± 6.3, *p* < 0.001), higher fasting glucose (151 ± 66 vs. 144 ± 63 mg/dL, *p* = 0.005), higher urine protein positivity (22.7% vs. 14.6%, *p* < 0.001), and higher fibrinogen levels (367 ± 156 vs. 347 ± 122 mg/dL, *p* = 0.002) (Table 1). In the multiple regression analysis of the total population, higher fibrinogen levels were not significantly associated with END after adjusting for confounders (*p* = 0.195) (Table 2). The interaction between diabetes status and fibrinogen levels regarding END was not statistically significant in the entire cohort (*p* = 0.101).

**Table 1:** Comparison of baseline data in patients with acute ischemic stroke admitted within 72 h

	END (*n* = 661)	Non-END (*n* = 3153)	P
Age	65.3 ± 12.6	63.5 ± 13.5	0.001
Male sex	409 (61.9%)	2017 (64.0%)	0.309
Hypertension	462 (70.0%)	2052 (65.8%)	0.039
Diabetes mellitus	262 (39.6%)	1098 (34.8%)	0.019
Initial NIHSS	8.2 ± 6.7	5.6 ± 6.3	<0.001
Discharge NIHSS	10.5 ± 10.3	3.2 ± 5.4	<0.001
TOAST			<0.001
Cardioembolism	160 (24.2%)	718 (22.8%)	
Atherosclerosis	201 (30.4%)	818 (25.9%)	
Small artery disease	159 (24.1%)	614 (19.5%)	
Others	141 (21.3%)	1003 (31.8%)	
Fasting glucose (mg/dL)	151 ± 66	144 ± 63	0.005
Glycated hemoglobin (%)	6.65 ± 1.57	6.46 ± 1.36	0.051
Urine protein positivity	100/441 (22.7%)	276/1896 (14.6%)	<0.001
Fibrinogen (mg/dL)	367 ± 156	347 ± 122	0.002
ESR (mm)	18.15 ± 17.09	17.57 ± 17.01	0.439
CRP (mg/dL)	0.81 ± 2.00	0.73 ± 2.33	0.459
BMI	23.8 ± 3.2	24.0 ± 3.4	0.163
Metabolic syndrome	288 (43.6%)	1295 (41.1%)	0.236
Lipid panel			
T.chol (mg/dL)	179.54 ± 42.07	179.15 ± 51.46	0.857
LDL (mg/dL)	107.42 ± 37.48	105.68 ± 35.45	0.262
HDL (mg/dL)	45.65 ± 12.87	45.94 ± 13.40	0.616
TG (mg/dL)	139.77 ± 122.26	142.83 ± 117.81	0.552

*END* early neurological deterioration, *NIHSS* National Institutes of Health Stroke Scale, *TOAST* trial of Org 10,172 in acute stroke treatment, *ESR* erythrocyte sedimentation rate, *CRP* c-reactive protein, *BMI* body mass index, *T.chol* total cholesterol, *LDL* low density lipoprotein, *HDL* high density lipoprotein, *TG* triglyceride

**Table 2:** A logistic regression model including potential factors associated with early neurologic deterioration in patients with acute ischemic stroke admitted within 72 h

	Odds ratio (95% confidence interval)	P
Age	1.00 (1.00–1.01)	0.605
Male sex	0.94 (0.78–1.14)	0.551
Hypertension	1.14 (0.93–1.40)	0.194
Diabetes mellitus	1.11 (0.92–1.33)	0.268
Initial NIHSS	1.07 (1.05–1.08)	<0.001
TOAST		<0.001
Cardioembolism	Reference	
Atherosclerosis	1.38 (1.07–1.78)	0.013
Small artery disease	1.97 (1.48–2.63)	<0.001
Others	0.94 (0.71–1.24)	0.652
Fibrinogen (mg/dL)		0.195
0–300 mg/dL	Reference	
300–600 mg/dL	1.20 (0.97–1.49)	0.091
> 600 mg/dL	1.36 (0.78–2.37)	0.195

*NIHSS* National Institutes of Health Stroke Scale, *TOAST* trial of Org 10,172 in acute stroke treatment

There were 1360 diabetic and 2454 non-diabetic patients. In the DM population (END = 262, non-END = 1098), a diagnosis of hypertension (81.6% vs. 74.6%, *p* = 0.017), initial NIHSS (10.4 ± 10.6 vs. 3.6 ± 5.7, *p* < 0.001), urine protein positivity (35.1% vs. 21.2%, *p* < 0.001), and fibrinogen levels (392.9 ± 204.2 mg/dL, *p* = 0.012) were significantly higher in the END population versus the non-END population (Table 3). Propensity score matching was performed for age, sex, initial NIHSS, and TOAST classification, with a relative multivariate imbalance measure L1 of 0.621 before matching, and 0.609 after matching. No covariate exhibited a large imbalance. After matching, proteinuria (34.7% vs. 23.4%, *p* = 0.007, and hyperfibrinogenemia (392.8 ± 204.6 vs. 361 ± 123.1 mg/dL, *p* = 0.009) were still significantly associated with END (Table 3). In the propensity score-matched population, further logistic regression analysis adjusting for age, sex, hypertension, TOAST classification, and positive proteinuria was done, and fibrinogen was significantly associated with END in the DM population (fibrinogen <300 mg/dL as reference; fibrinogen levels 300 ~ 599 mg/dL, odds ratio: 1.618, 95% confidence interval: 1.037–2.525, *p* = 0.034, fibrinogen levels ≥600 mg/dL, 2.575, 1.018–6.514, *p* = 0.046) (Table 4).

**Table 3:** Comparison before and after propensity score matching^a^ in patients with diabetes and acute ischemic stroke admitted within 72 h

	Before matching			After matching		
	END (*n* = 262)	Non-END (*n* = 1098)	P	END (*n* = 261)	Non-END (*n* = 522)	P
Age	65.3 ± 11.8	65.1 ± 11.7	0.771	65.3 ± 11.8	64.8 ± 11.8	0.593
Male sex	160 (61.1%)	700 (63.8%)	0.418	159 (60.9%)	315 (60.3%)	0.877
Hypertension	213 (81.6%)	812(74.6%)	0.017	213 (81.6%)	431 (82.6%)	0.741
Initial NIHSS	8.0 ± 6.6	5.7 ± 6.4	<0.001	7.9 ± 6.6	7.6 ± 6.8	0.562
Discharge NIHSS	10.4 ± 10.6	3.6 ± 5.7	<0.001	10.4 ± 10.5	4.8 ± 6.5	<0.001
TOAST			0.167	76 (23.4%)		0.169
Cardioembolism	52 (19.8%)	221 (20.1%)		52 (19.9%)	111 (21.3%)	
Atherosclerosis	88 (33.6%)	328 (29.8%)		88 (33.7%)	163 (31.2%)	
Small artery disease	64 (24.4%)	235 (21.4%)		64 (24.5%)	102 (19.5%)	
Others	58 (22.1%)	314 (28.6%)		57 (21.8%)	146 (28.0%)	
Fasting glucose (mg/dL)	190.9 ± 84.1	184.0 ± 85.5	0.238	190.7 ± 84.2	189.5 ± 87.8	0.852
Glycated hemoglobin (%)	7.5 ± 1.7	7.3 ± 1.5	0.119	7.5 ± 1.7	7.4 ± 1.6	0.392
Urine protein positivity	59 (35.1%)	143 (21.2%)	<0.001	58 (34.7%)	76 (23.4%)	0.007
Fibrinogen (mg/dL)	392.9 ± 204.2	359.0 ± 119.1	0.012	392.8 ± 204.6	361.1 ± 123.1	0.009
ESR (mm)	22.0 ± 20.5	21.2 ± 20.1	0.547	22.0 ± 20.5	21.3 ± 18.9	0.643
CRP (mg/dL)	0.9 ± 2.3	0.9 ± 2.8	0.931	0.9 ± 2.3	1.1 ± 3.2	0.528
BMI				24.2 ± 3.3	24.5 ± 3.2	0.317
Metabolic syndrome	149 (56.9%)	609 (55.5%)	0.681	149 (57.1%)	305 (58.4%)	0.720
Lipid panel						
T.chol (mg/dL)	179.6 ± 43.2	181.7 ± 69.0	0.629	179.3 ± 43.0	182.2 ± 47.4	0.396
LDL (mg/dL)	106.3 ± 37.7	106.1 ± 37.4	0.919	106.0 ± 37.4	107.5 ± 38.1	0.604
HDL (mg/dL)	43.7 ± 11.7	43.9 ± 15.0	0.825	43.7 ± 11.7	44.0 ± 12.1	0.775
TG (mg/dL)	158.6 ± 113.5	163.4 ± 133.4	0.600	158.7 ± 113.7	165.6 ± 153.3	0.521

*END* early neurological deterioration, *NIHSS* National Institutes of Health Stroke Scale, *TOAST* trial of Org 10,172 in acute stroke treatment, *ESR* erythrocyte sedimentation rate, *CRP* c-reactive protein, *BMI* body mass index, *T.chol* total cholesterol, *LDL* low density lipoprotein, *HDL* high density lipoprotein, *TG* triglyceride

^a^ Age, sex, initial NIHSS and TOAST were adjusted

**Table 4:** A logistic regression model for END after propensity score matching^a^ in patients with diabetes mellitus admitted within 72 h of acute ischemic stroke

	Odds ratio (95% confidence interval)	P
Age	1.01 (0.99–1.03)	0.397
Male sex	1.22 (0.80–1.87)	0.352
Hypertension	1.01 (0.584–1.74)	0.982
Initial NIHSS	1.01 (0.98–1.05)	0.434
TOAST		0.098
Cardioembolism	Reference	
Atherosclerosis	1.24 (0.71–2.16)	0.445
Small artery disease	2.01 (1.00–4.02)	0.049
Others	0.95 (0.51–1.75)	0.858
Positive urine proteins	1.40 (0.90–2.17)	0.140
Fibrinogen per 300 mg/dl		0.044
0–300 mg/dL	Reference	
300–600 mg/dL	1.62 (1.04–2.53)	0.034
> 600 mg/dL	2.60 (1.02–6.51)	0.046

*END* early neurological deterioration, *NIHSS* National Institutes of Health Stroke Scale, *TOAST* trial of Org 10,172 in acute stroke treatment

^a^ Age, sex, initial NIHSS and TOAST were adjusted

In the non-DM population (END = 399, Non-END = 2055), age (65.3 ± 13.2 vs. 62.7 ± 14.3, *p* < 0.001), initial NIHSS (8.4 ± 6.8 vs. 5.6 ± 6.3, *p* < 0.001), and fasting glucose levels (125.6 ± 30.9 vs. 122.1 ± 29.7 mg/dL, *p* = 0.036) were significantly higher in the END group, as were differences in TOAST classification (*p* < 0.001) (Additional file 2: Table S1). Propensity score matching was performed for age, sex, initial NIHSS, and TOAST classification, with a relative multivariate imbalance measure L1 of 0.581 before matching, and 0.525 after matching. No covariate exhibited a large imbalance. After matching, TOAST classification (*p* = 0.003), elevated total cholesterol (179.5 ± 41.3 vs. 173.6 ± 38.4 mg/dL, *p* = 0.015), and elevated low density lipoprotein (108.2 ± 37.4 vs. 102.8 ± 34.3 mg/dL, *p* = 0.015) were significantly associated with END (Additional file 2: Table S1). In further regression analysis in the matched population, including age, male sex, presence of hypertension, initial NIHSS, TOAST classification, and total cholesterol levels as covariables, fibrinogen did not prove to be associated with END (*p* = 0.393) (Additional file 2: Table S2).

## Discussion

The results of this study suggest that hyperfibrinogenemia in patients with acute stroke and DM is associated with END, using both propensity score matching and multiple logistic regression analysis. Whereas the influence of hyperfibrinogenemia on END was consistently seen in the DM subgroup, it disappeared in the non-DM subgroup.

Hyperfibrinogenemia itself may directly induce a higher frequency of END in diabetic individuals through activation of the coagulation cascade. Fibrinogen forms fibrin clots as part of the final common stages of both the extrinsic and intrinsic pathways of the coagulation cascade [19]. Atherogenesis and the growth of atheromatous lesions are also initiated in part by fibrin deposition [20, 21]. Fibrinogen also facilitates platelet aggregation by binding to the glycoprotein IIb/IIIa receptor, increasing its reactivity [22].

Hyperfibrinogenemia may also indirectly reflect a prothrombotic condition induced by hyperinsulinemia and prolonged glycation. Levels of fibrinogen in diabetic patients correlate with insulin resistance and metabolic syndrome [13]. Prolonged glycation also induces a prothrombotic condition. Studies on fibrin clot structure under diabetic conditions have shown fibrinogen to be glycated in vivo [23], causing a change in the fibrin clot structure that reduces permeability [24], decreases fibrinolysis [25], and decreases α-chain crosslinking [26]. Thus, the effects of insulin resistance and prolonged glycation increase the risk of thrombosis, which underpins the development of vascular disease [27]. These studies lead us to assume that fibrinogen levels may represent a marker of platelet aggregation or progression to a prothrombotic phenotype in patients with diabetes [27].

This is the first study to link diabetic hyperfibrinogenemia to a higher frequency of END after ischemic stroke. Epidemiological studies have established that elevated fibrinogen levels are strongly and independently correlated with the risks of coronary arterial disease (CAD), stroke, and peripheral arterial disease [16, 28–31]. In stroke, carotid artery stenosis is significantly associated with elevated fibrinogen levels [32], and placebo data analysis in the Stroke Treatment with Ancrod Trial (STAT) and European Stroke Treatment with Ancrod Trial (ESTAT) has shown that plasma fibrinogen levels at stroke onset are independently associated with a poor functional outcome [33]. However, it has not been reported that hyperfibrinogenemia in patients with diabetes is associated with END in the acute stroke period.

It is conceivable that stronger antiplatelet treatments might be needed for patients with diabetes and hyperfibrinogenemia following acute ischemic stroke. Of note, elevated fibrinogen levels in our group of patients with diabetes may be linked to antiplatelet resistance, which may in turn result in END. Previous large scale trials, such as the Japanese Primary Prevention of Atherosclerosis with Aspirin for Diabetes (JPAD) trial [34], and the Prevention of Progression of Arterial Disease and Diabetes (POPAPAD) trial [35], have shown low efficacy of aspirin monotherapy for prevention of cardiovascular events in patients with diabetes. In addition, recent data show that responses to clopidogrel in patients with diabetes are suboptimal [36]. Such data suggest that diabetes is associated with considerable resistance to aspirin and clopidogrel therapy, and such insufficient antiplatelet responses could be an underlying causal factor for the frequent END observed in this population. In accordance with the elevated fibrinogen levels in our data, a recent study evaluating platelet aggregation characteristics in acute coronary syndrome found antiplatelet drug resistance to be significantly associated with metabolic syndrome, and fibrinogen levels and high sensitivity-CRP were higher in this population [37]. Serum fibrinogen levels appeared to be strongly associated with drug resistance [37]. In this case, elevated fibrinogen levels may call for more radical choices for antiplatelet therapy in such a population.

Apart from the antiplatelet issue, the idea that reduced fibrin, which is formed by fibrinogen in blood, may reduce blood viscosity, improve blood flow, and help remove the blood clot blocking the artery and re-establish blood flow has inspired clinical trials testing the therapeutic effects of the fibrin-depleting agents ancrod and defibrase [38]. In eight trials involving 5701 patients, fibrin-depleting agents marginally reduced the proportion of patients who were dead or disabled at the end of follow-up, and there were fewer stroke recurrences in the treatment group than in the control group. However, symptomatic intracranial hemorrhage was about twice as common in the treatment group compared with the control group [38]. The use of fibrin-depleting agents to reduce blood viscosity in patients with DM and elevated fibrinogen at risk for END holds similar problems. Plasma fibrinogen levels are associated with silent cerebrovascular lesions [39], and although this relationship may be evidence that increased viscosity by fibrinogen is a risk factor for stroke, deep white matter hyperintensities, on the other hand, are a known risk factor for intracerebral hemorrhage [40]. Accordingly, the use of fibrin-depleting agents in patients with DM may confer a higher risk of hemorrhagic complications. Thus, while the selective use of fibrin-depleting agents in patients with DM and END may be considered, further clinical studies to address the risks are needed prior to making such treatment a recommendation.

We acknowledge that our study has limitations. First, due to the retrospective nature of the study, there may be selection bias. We tried to minimize this issue by maximizing the number of patients included in the study and including adequate propensity score matching. Second, our somewhat long temporal definition of END (<7 days) incorporates a range of heterogeneous underlying mechanisms, and different definitions are used in other reports. Due to the retrospective nature and large number of enrolled patients in the study, the heterogeneity of END could not be addressed in this study. Third, because of the focus on lab data in this study, medication factors and imaging variables such as intracranial occlusion, stenosis, or diffusion weighted image volume were not included for analysis. However, variables that may have similar implications such as TOAST classification, and NIHSS scores were included, and the large patient numbers included may supplement such limitations. We hope to address these issues in later prospective studies.

## Conclusion

In conclusion, hyperfibrinogenemia at admission in patients with acute ischemic stroke and DM is independently associated with END. This finding suggests that an elevated fibrinogen level is a marker of a prothrombotic or antiplatelet-resistant condition related to DM that could affect the patient’s prognosis. On the other hand, these results also reveal a category of patients wherein fibrin-depleting therapy could be significantly more effective.

## Additional files

Additional file 1: Figure S1. A dot plot of standardized mean differences before and after propensity score matching in (A) DM population and (B) non-DM population. (TIFF 261 kb)

Additional file 2: Table S1. Comparison before and after propensity score matching in patients with acute ischemic stroke admitted within 72 h, but without diabetes. **Table S2.** A logistic regression model for END after propensity score matching in patients without diabetes mellitus admitted within 72 h of acute ischemic stroke. (DOCX 25 kb)

## Abbreviations

CAD: Coronary arterial disease
CRP: C-reactive protein
DM: Diabetes mellitus
END: Early neurological deterioration
ESR: Erythrocyte sedimentation rate
FDP: Fibrin degradation products
HbA1c: Glycated hemoglobin
NIHSS: National Institute of Health Stroke Scale
TOAST: Trial of Org 10,172 in acute stroke treatment
