# Supplementary figures and images for: Association of fibrinogen level with early neurological deterioration among acute ischemic stroke patients with diabetes
Source: BMC Neurol. 2017 May 19;17:101. doi: 10.1186/s12883-017-0865-7 (PMC5438529; doi:10.1186/s12883-017-0865-7)

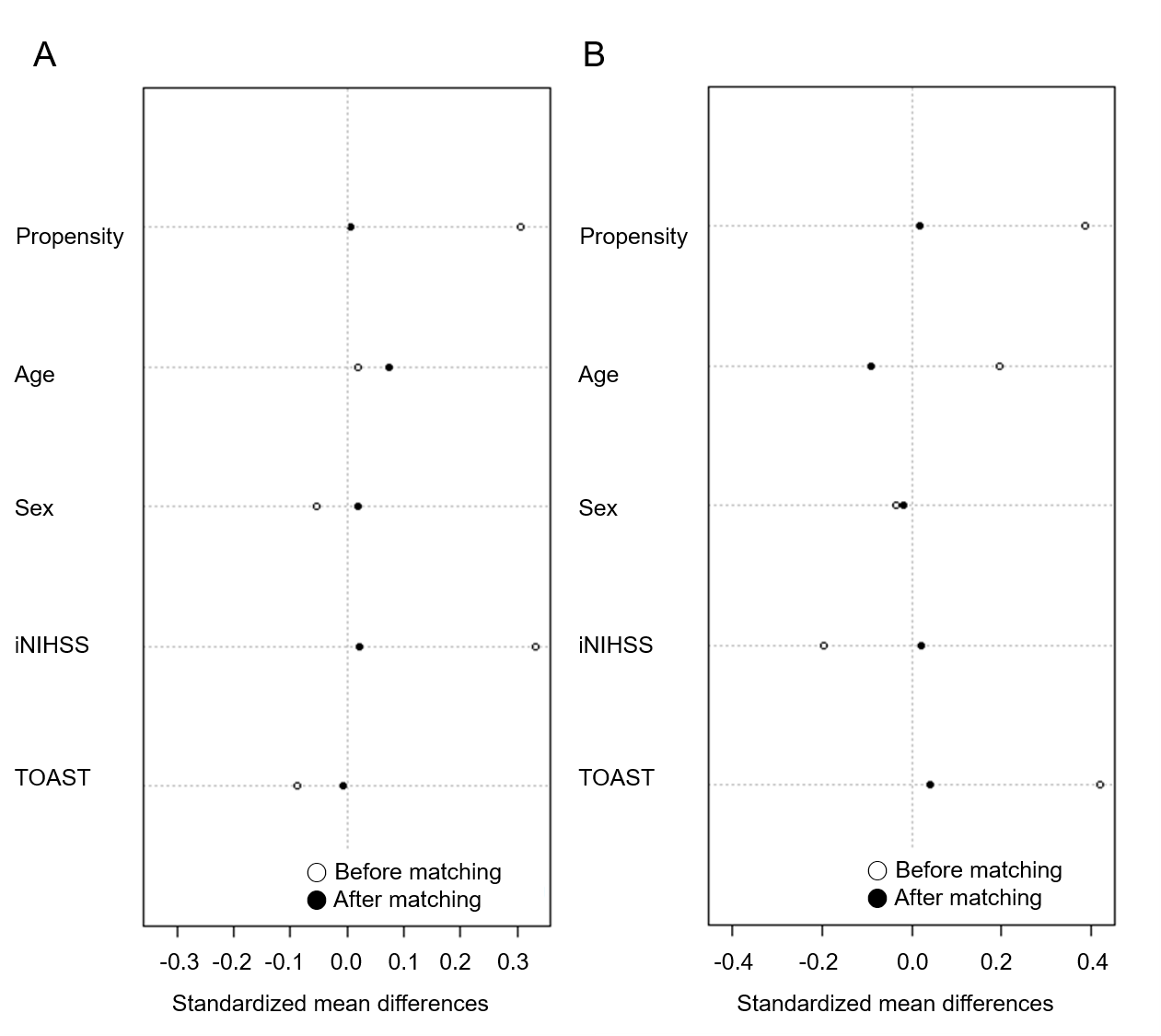

Supplement: Supplementary file 1 — A dot plot of standardized mean differences before and after propensity score matching in (A) DM population and (B) non-DM population. (TIFF 261 kb) [file 12883_2017_865_MOESM1_ESM.tif]
