# Supplementary material for: Association of fibrinogen level with early neurological deterioration among acute ischemic stroke patients with diabetes
Source: BMC Neurol. 2017 May 19;17:101. doi: 10.1186/s12883-017-0865-7 (PMC5438529; doi:10.1186/s12883-017-0865-7)
Supplement: Supplementary file 2 — Comparison before and after propensity score matching in patients with acute ischemic stroke admitted within 72 h, but without diabetes. Table S2. A logistic regression model for END after propensity score matching in patients without diabetes mellitus admitted within 72 h of acute ischemic stroke. (DOCX 25 kb) [file 12883_2017_865_MOESM2_ESM.docx]

**ASSOCIATION OF FIBRINOGEN LEVEL WITH EARLY NEUROLOGICAL DETERIORATION AMONG ACUTE ISCHEMIC STROKE PATIENTS WITH DIABETES**

Seong-Joon Lee, MD, MS,^1^ Ji Man Hong, MD, PhD,^1^ Sung Eun Lee, MD,^1^ Jung-Dong Lee, MS,^2^ Dae Ryong Kang, PhD,^2^ Bruce Ovbiagele, MD, FRCP,^3^ Andrew M. Demchuk, MD, FRCPC, ^4^ Jin Soo Lee, MD, PhD^1^

1. Department of Neurology, Ajou University School of Medicine, Ajou University Medical Center, Suwon, South Korea.

2. Office of Biostatistics, Ajou University School of Medicine, Suwon, South Korea

3. Department of Neurology, Medical University of South Carolina, Charleston, South Carolina, USA

4. Calgary Stroke Program, Departments of Clinical Neurosciences and Radiology, Hotchkiss Brain Institute, University of Calgary, Calgary, AB, Canada

**Corresponding author**: Jin Soo Lee, MD, PhD

Department of Neurology,

Ajou University School of Medicine,

San 5, Woncheon-dong, Yeongtong-gu,

Suwon, Kyungki-do, 443–721, South Korea

Phone: +82-31-219-5175

Fax: +82-31-219-5178

E-mail: [jinsoo22@gmail.com](mailto:jinsoo22@gmail.com)

**Table S1**. Comparison before and after propensity score matching^*^ in patients with acute ischemic stroke but without diabetes, admitted within 72 hours.

|  | Before matching | | | After matching | | |
| --- | --- | --- | --- | --- | --- | --- |
|  | END (n=399) | Non-END (n=2055) | P | END (n=399) | Non-END (n=798) | P |
| Age | 65.3±13.2 | 62.7±14.3 | <0.001 | 65.3±13.2 | 66.4±13.4 | 0.145 |
| Male sex | 249 (62.4%) | 1317 (64.1%) | 0.522 | 249 (62.4%) | 505 (63.3%) | 0.767 |
| Hypertension | 249 (62.4%) | 1240 (61.1%) | 0.636 | 249 (62.4%) | 494 (62.6%) | 0.945 |
| Initial NIHSS | 8.4±6.8 | 5.6±6.3 | <0.001 | 8.4±6.8 | 8.2±6.7 | 0.520 |
| Discharge NIHSS | 10.6±10.2 | 3.0±5.2 | <0.001 | 10.6±10.2 | 4.4±6.2 | <0.001 |
| TOAST |  |  | <0.001 |  |  | 0.003 |
| Cardioembolism | 108 (27.1%) | 497 (24.2%) |  | 108 (27.1%) | 246 (30.8%) |  |
| Atherosclerosis | 113 (28.3%) | 490 (23.8%) |  | 113 (28.3%) | 216 (27.1%) |  |
| Small artery disease | 95 (23.8%) | 379 (18.4%) |  | 95 (23.8%) | 126 (15.8%) |  |
| Others | 83 (20.8%) | 689 (33.5%) |  | 83 (20.8%) | 210 (26.3%) |  |
| Fasting glucose (mg/dL) | 125.6±30.9 | 122.1±29.7 | 0.036 | 125.6±30.9 | 123.6±32.6 | 0.332 |
| Glycated hemoglobin (%) | 5.7±0.3 | 5.7±0.3 | 0.209 | 5.7±0.3 | 5.6±0.3 | 0.211 |
| Urine protein positivity | 41 (15.0%) | 133 (10.9%) | 0.055 | 41 (15.0%) | 62 (12.6%) | 0.348 |
| Fibrinogen (mg/dL) | 350.3±110.2 | 340.6±123.1 | 0.160 | 350.3±110.2 | 352.0±152.4 | 0.850 |
| ESR (mm) | 15.6±13.9 | 15.7±14.8 | 0.956 | 15.6±13.9 | 16.8±15.7 | 0.196 |
| CRP (mg/dL) | 0.7±1.8 | 0.6±2.0 | 0.324 | 0.7±1.8 | 0.8±2.4 | 0.777 |
| BMI |  |  |  |  |  |  |
| Metabolic syndrome | 139 (34.8%) | 686 (33.4%) | 0.573 | 139 (34.8%) | 265 (33.2%) | 0.574 |
| Lipid panel |  |  |  |  |  |  |
| T.chol (mg/dL) | 179.5±41.3 | 177.8±38.9 | 0.416 | 179.5±41.3 | 173.6±38.4 | 0.015 |
| LDL (mg/dL) | 108.2±37.4 | 105.5±34.4 | 0.193 | 108.2±37.4 | 102.8±34.3 | 0.015 |
| HDL (mg/dL) | 47.0±13.4 | 47.0±12.3 | 0.900 | 47.0±13.4 | 46.3±12.1 | 0.435 |
| TG (mg/dL) | 127.4±123.3 | 131.8±106.9 | 0.474 | 127.4±123.3 | 125.5±94.9 | 0.780 |

END, Early neurological deterioration; NIHSS, National Institutes of Health Stroke Scale; TOAST, Trial of Org 10172 in Acute Stroke Treatment; ESR, Erythrocyte Sedimentation Rate; CRP, C-Reactive Protein; BMI, Body Mass Index; T.chol, Total Cholesterol; LDL, Low Density Lipoprotein; HDL, High Density Lipoprotein; TG, Triglyceride.

* Age, sex, initial NIHSS and TOAST were adjusted.

**Table S2**. A logistic regression model for END after propensity score matching^*^ in patients without diabetes mellitus admitted within 72 hours of acute ischemic stroke.

|  | Odds ratio (95% confidence interval) | P |
| --- | --- | --- |
| Age | 0.99 (0.98 – 1.00) | 0.102 |
| Male sex | 0.91 (0.69– 1.21) | 0.528 |
| Hypertension | 1.02 (0.78 – 1.34) | 0.870 |
| Initial NIHSS | 1.030 (1.01 – 1.05) | 0.017 |
| TOAST |  | 0.004 |
| Cardioembolism | Reference |  |
| Atherosclerosis | 1.10 (0.77 – 1.60) | 0.606 |
| Small artery disease | 1.82 (1.20 – 2.75) | 0.005 |
| Others | 0.89 (0.61 – 1.31) | 0.566 |
| Total cholesterol level | 1.00 (1.00 –1.01) | 0.054 |
| Fibrinogen per 300 mg/dl |  | 0.393 |
| 0 – 300 mg/dL | Reference |  |
| 300 – 600 mg/dL | 1.05 (0.80 – 1.39) | 0.706 |
| > 600 mg/dL | 0.56 (0.21 – 1.43) | 0.554 |

END, Early neurological deterioration; NIHSS, National Institutes of Health Stroke Scale; TOAST, Trial of Org 10172 in Acute Stroke Treatment.

* Age, sex, initial NIHSS and TOAST were adjusted.
